# The collective dynamics of frustrated biological neuron networks

**DOI:** 10.21203/rs.3.rs-4006823/v1

**Published:** 2024-04-05

**Authors:** Guanyu Li, Ryan LeFebre, Alia Starman, Patrick Chappell, Andrew Mugler, Bo Sun

**Affiliations:** 1 Oregon State University, Department of Physics, Corvallis, 97331, USA; 2 Department of Physics and Astronomy, University of Pittsburgh, Pittsburgh, PA 15260; 3 Department of Biomedical Sciences, Carlson College of Veterinary Medicine, Oregon State University, Corvallis, OR 97331

## Abstract

To maintain normal functionality, it is necessary for a multicellular organism to generate robust responses to external temporal signals. However, the underlying mechanisms to coordinate the collective dynamics of cells remain poorly understood. Here we study the calcium activity of micropatterned biological neuron networks excited by periodic ATP stimuli. Combining quantitative experiments, physical and biological manipulation of cells, as well as mathematical modeling, we show that isolated cells in a network become more synchronized at longer period of stimuli through noise cancellation. However, slowly varying external signal also increases gap junction coupling between connected nodes in the network; and gap junction mediated communication may destroy network synchronization due to special nonlinear bifurcations exhibited by the excitable dynamics of neuronal cells. Based on our results, we propose that a biological neuron network supported by gap junctional communication encodes external temporal signals in its network dynamics. A sparely connected network approaches synchronization as input signal slows down, whereas a highly connected network enters dynamic frustration in the same situation.

## Introduction

As a key task to sustain normal functionality, communities of living systems and multicellular organisms must produce coordinated responses to temporal signals from the environment^12^. The period of the environment stimuli may vary from seconds, such as in heart beats^3^, to hours, such as in mammalian circadian rhythm^4^. Unraveling the mechanisms that coordinate the individual responses to generate robust collective dynamics hold the key to understand the organizational principles in biology^56^.

Synchronization is a prevalent form of collective sensory dynamics to temporal signals, and is observed in vast array of living systems such as bacteria colonies^7^, social amoeba^8^, mammalian muscles^9^, and in the human brain^10^. For a multicellular system to synchronize, it relies on robust intercellular communication that couples cells through mechanical^11^, chemical^12^ or electrical dialogues^13^ in order to balance against the intrinsic and extrinsic noises^14^. However, uncontrolled synchronization is not always desirable and can sometime be fatal^15^. The fundamental question of how the same type of intercellular coupling can both promote and limit synchronization remains poorly understood.

In our previous studies, we find various cell types, including fibroblasts^16^, endothelial cells^17^ and neural cells^18^, all exhibit collective sensory responses to temporal signals, orchestrated through gap junctional communications among individual cells. In particular, the communication strength between cells is modulated by the period of external stimuli, and the information transfer between cells is minimized at complete synchronization^18,17^. Thus, gap junctions serve as dynamic and adaptable communication channels. When a multicellular organism depends on gap junctions to integrate the nonlinear dynamics of individual cells, as in the case of a biological neural network, it gives rise to complex network dynamics that remains incompletely characterized^19,20^.

In this study we combine quantitative experiments and computational modeling to investigate the synchronization of communicating neuronal cells driven by periodic extracellular ATP (Adenosine triphosphate) stimuli. We examine the calcium dynamics of KTaR cells, a neuronal cell line we derived from KNDy (Kisspeptin, neurokinin B, and dynorphin) neurons within the arcuate nucleus of an adult female mouse^21^. We control the communication between KTaR cells physically through cell-adhesive micropatterning, and biologically through CRISPR-Cas9 knockout of gap junction-forming connexin proteins. We show that the level of synchronization strongly depends on the period of ATP stimuli, as well as the connectivity between cells. Surprisingly, we find gap junctional communication between cells may destroy synchronization, resulting in a dynamically frustrated neuronal network. Our theoretical model suggests that nonlinear bifurcation may provide key insights in understanding the experimental observations. Together, we reveal that biological neural networks display a wide array of complex collective behaviors. These behaviors are primarily influenced by external temporal signals and are further modulated by internal communications through gap junctions.

## Results

**Figure 1.**
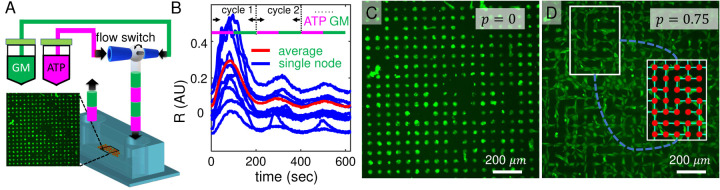
Micropatterned biological neuron networks (MBNNs) respond to temporal stimuli that trigger intracellular calcium dynamics. (A) schematic of the experimental set-up. (B) Fluorescent intensity of calcium imaging for individual node (Blue) and population average (Red). In this example external stimuli varies between growth medium (GM) and adenosine triphosphate (ATP) at a period of 200 seconds. (C) A sample fluorescent calcium image of a MBNN showing the micropatterned KTaR cell monolayer. In this example the network is disconnected with connectivity *p* = 0. Each node has a radius of 20 *μ*m and the edge-to-edge separation between nodes is 30 *μ*m. (D) A sample fluorescent calcium image of a MBNN with connectivity *p* = 75%. The nodes are bridged by micropatterned KTaR cells with the bridge width equals to 10 *μ*m. Inset: We use automated image registration to identify effective nodes and bridges so that regions without cells attached are excluded.

In order to study the collective response to external stimuli by neuronal networks with precisely controlled spatial connectivity, we incorporate micropatterned biological neuron networks (MBNNs) to a microfluidics device (Fig. 1). In particular, the microfluidics consists of a computer-interfaced flow switch to deliver alternating growth media and ATP solution to the cell monolayer with periods ranging from 40 seconds to 200 seconds (Fig. 1A), and with duty cycle of 50% as described previously^18^. A substrate is patterned by coating a glass slide of fibrinogen surrounded by PEG (polyethylene glycol) using photolithography (see SI section 1a and 1b). Fibrinogen promotes and PEG prohibits cell adhesion. We preload KTaR cells with fluorescent calcium indicator, then pattern the cells into circular nodes of radius 20 μm. Each node typically consists of 2–3 cells. When exposed to periodic ATP stimuli, we measure the node response *R*_*i*_(*t*) by computing the average fluorescent intensity of a node *i* compared to its basal value (Fig. 1B). To modify the spatial connectivity of the MBNNs, we arrange the nodes into a square lattice and set the edge-to-edge distance between nearest nodes to be 30 μm. Between nearby nodes, a line of single cell width is patterned to connect the node pair at probability *p*. When *p* = 0, all nodes are isolated (Fig. 1C). When *p* = 0.75, each node is on average connected to three of its four neighbors (Fig. 1D). We notice that at larger connectivity values cells may overgrow beyond the designed fibrinogen regions, possibly due to the resolution of our photolithography process. Therefore to ensure accurate spatial patterning in the study, we keep the maximum connectivity to be *p* =0.75.

**Figure 2.**
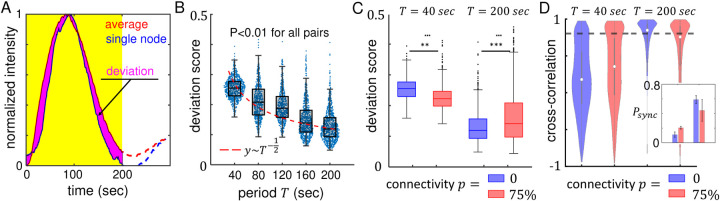
Micropatterned biological neuron networks (MBNNs) demonstrate synchronized responses to temporal stimuli with the degree of synchronization modulated by the network connectivity and period of driving signal. (A) We define deviation score to quantify the phase difference between the calcium dynamics of individual node and the population average. Due to the uncertainty of stimuli arrival time, we compute the deviation score in a moving window of size equal to the period and average results from the moving windows. Within the moving window, all responses are first linearly scaled to range [0,1]. Then deviation score is calculated as the ratio between the area of magenta portion (labeled as deviation in (A)) to the area of yellow rectangle. (B) At connectivity *p* = 0, the deviation score decreases monotonically at larger period *T*, indicating better synchronization when the driving signal varies slowly. For each period, 600–1000 data points are included from at least 3 replicating experiments. Red line shows an approximate $T^{-\frac{1}{2}}$ scaling. (C) The deviation scores at two driving periods (*T* = 40 seconds and *T* = 200 seconds) with network connectivity equals to 0 or 75%. The results show node-to-node communication facilitates synchronization at short period but destroys synchronization at long period. (D) Cross-correlation analysis between neighboring nodes show consistent results as in (C). Here a pair of nodes are considered as neighbors if they are bridged by cells. For disconnected networks neighboring nodes are randomly sampled nearest nodes in space. At *T* = 40 seconds, neighboring nodes of highly connected networks have greater cross-correlation values than those of a disconnected network. At *T* = 200 seconds, neighboring nodes of highly connected networks are less correlated than those of a disconnected network. We empirically consider nodes with cross-correlation greater than 0.8 as well-synchronized. The threshold is indicated as the dashed line in (D). In (C-D) each group includes 600–1000 data points from at least 3 replicating experiments. Inset: fraction of well-synchronized node pairs among all neighboring pairs (*P*_*sync*_). Errorbars in the inset are standard deviations from three replicating experiments. In (B-C), the lines and boxes show the median, upper and bottom quartiles. In (D) the circles and lines show the median, upper and bottom quartiles.

To characterize the synchronicity of the responses of individual nodes, we compute two quantities: the deviation score between the responses of individual nodes and the the population average, and the cross-correlation coefficient between neighboring nodes. As shown in Fig. 2A, the deviation score of a node is defined by first obtaining the normalized response *R*_*i*,*N*_(*t*) in a moving window of width equal to the period *T* of the stimuli, then calculating the area (absolute value) between the curves *R*_*i*,*N*_ and ⟨*R*_*i*,*N*_⟩_*population*_. Here ⟨*R*_*i*,*N*_⟩_*population*_ represents average over all nodes within the field of view. To ensure fair treatment for different periods, we divide the area between the curves by the period *T*, thereby measuring the deviation per unit time. The deviation score is a dimension-less quantity that approaches zero when all nodes perfectly synchronize.

We find that deviation score continuously decreases at longer period *T* even when the nodes are isolated (*p* = 0), suggesting that cells respond more uniformly to slowly varying external stimuli (Fig. 2B). The trend also holds when the nodes are connected, as shown in Fig. 2C. Because the observation is independent of node-node communication, we attribute the synchronized response at longer period to a decreasing impact of intercellular heterogeneity. Specifically, suppose the nodes are randomly activated during the stimuli ON cycle, the time delay *δt* of the node to the external stimuli would follow a Poisson distribution of variance *σ*^2^ ~ *T*. Then the average deviation score would scale as $\frac{\sigma }{T}$, or $\frac{1}{\sqrt{T}}$. The scaling agrees qualitatively to the experiment observation in Fig. 2B (red curve), and quantitatively to our theoretical model to be detailed later.

When external signal varies rapidly, such as the case of *T* = 40 seconds, intercellular communication moderately improves synchronization (Fig. 2C). This is consistent with the increased cross-correlation coefficients between neighboring nodes (Fig. 2D). However, when external signal varies slowly, such as the case of *T* = 200 seconds, MBNNs with higher connectivity exhibit asynchronous responses. Indeed at *T* = 200 sec, average deviation score increases by 20% when connectivity increases from *p* =0 to *p* =0.75. Concurrently, fractions of well-synchronized nearest pairs – neighboring nodes with cross-correlation coefficient higher than 0.8 – decreases from 70% to 55% (Fig. 2D inset). These results confirm an unexpected observation, that when driven by a slowly varying external signal the communication within a neuronal network curtails, rather than enhances synchronization. Therefore we find MBNNs to be dynamically frustrated, similar to a geometrically frustrated antiferromagentic system^22^.

**Figure 3.**
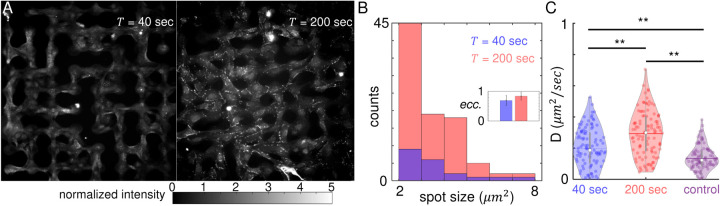
Temporal signal modulates the properties of gap junctions. (A) Immunofluorescent images of connexin 43 proteins of KTaR cells forming micropatterned biological neuron networks (MBNNs). The MBNNs are exposed to periodic ATP stimuli of period *T* = 40 seconds (left) and *T* = 200 seconds (right) before fixation and immunostaining. The image intensity has been normalized so that the background has a value of zero, cell cytoplasm has an mean value of 1 (see SI 2c for normalization procedures). (B) Spot analysis of immunofluorescent images after binarizing the images in (A) with a threshold of 1 (the average intensity of cytoplasm). The histogram shows the distribution of spot sizes. Inset: eccentricity of the spots. For circular spots, eccentricity is 0. For line spots, eccentricity is 1. (C) Effective intercellular diffusion coefficient *D* measured via FRAP (fluorescent recovery after photobleaching, see SI 2d for more details). The KTaR cell monolayers are exposed various conditions before FRAP measurements: periodic ATP stimuli of period *T* = 40 seconds (blue), of period *T* = 200 seconds (red), or zero concentration of ATP with continuous flow of growth media (control, purple). Five repeated experiments for each condition are conducted and the diffusion coefficients from 20 consecutive frames are computed. Statistical test: *N* = 100 for each group, and ANOVA test is used for comparison. **: *p* < 0.01.

To gain insights into the dynamics of MBNNs under temporal signals, we quantify the spatial profile of gap junctions by immunofluorescence of connexin 43. In particular, MBNNs are subject to periodic ATP stimuli for short (40 seconds) or long (200 seconds) periods for a total of 15 minutes. Immediately after the stimulation, MBNNs are fixed and stained using fluorescent antibodies (see SI 1e for additional details). To quantify the fluorescent signals, we normalize the images by first subtracting the background, then scaling the intensity such that the basal level intensity of cytoplasm is one (see SI section S2 for image analysis). The normalized immunofluorescent images shown in Fig. 3A clearly demonstrate the different gap junction expressions under rapid and slow temporal signals. When external signal varies rapidly (*T* = 40 sec), connexin 43 appears to be mostly diffusive throughout the cells. However, when external signal varies slowly (*T* = 200 sec), connexin 43 forms punctate plaques at the cellular junctions. Quantitative analysis of the bright spots further confirms the visual observations. At *T* = 200 seconds, connexin 43 assembles into more densely populated, more elongated structures compared with the case of *T* = 40 seconds (Fig. 3B, see SI Fig.6 for additional examples of immunofluorescent images).

In addition to morphological characterizations, we have also tested if the conductivity of gap junctions can be modulated by external temporal signals. To this end, we subject confluent monolayers of KTaR cells to periodic ATP stimuli and then measure the effective intercellular diffusion coefficients using fluorescent recovery after photobleaching (FRAP) approach we developed previously^16^ (see also SI section S2 for details). As shown in Fig. 3C the diffusion coefficient *D* of a cell monolayer depends strongly on the period of ATP stimuli. At *T* = 40 seconds, the average diffusion coefficient is approximately 0.2 *μ*^2^/*sec*. When the temporal signal slows down to *T* = 200 seconds, the average diffusion coefficient increases by 50% to 0.32 *μ*^2^/*sec*. As a control, we have also conducted FRAP experiments without ATP stimuli, such that cell monolayers are subject to constant flow of growth media. The resulted diffusion coefficient is moderately smaller compared with the case of rapid stimuli (*T* = 40 seconds), and is less than half of the value for slow stimuli (*T* = 200 seconds). Taken together, both morphological (Fig. 3A–B) and functional (Fig.3C) measurements confirm that intercellular communication is enhanced when external temporal signal slows down.

**Figure 4.**
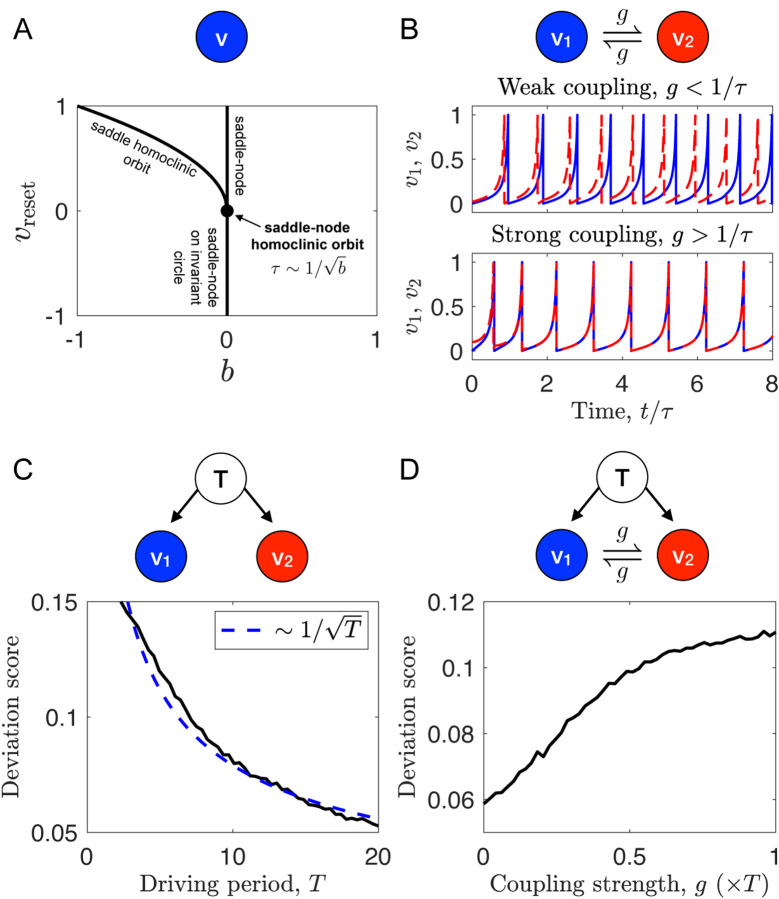
A computational model demonstrates the underlying mechanisms that lead a communicating neural network to be dynamically frustrated. (A) The quadratic integrate-and-fire model neuron exhibits a saddle-node homoclinic orbit (SNHO) bifurcation where other bifurcations meet^25^. (B) Without external driving, weak gap-junction coupling desynchronizes two neurons (top), whereas strong coupling synchronizes them (bottom). Here *τ* = 20, *v*_peak_ = 1, and *gτ* = 1/3 (top) or 3 (bottom). (C) With external driving but without gap-junction coupling, two neurons become more synchronized (lower deviation score) as the driving period increases. Here *g*_0_ = 0.1 and *v*_peak_ = 1. (D) With both external driving and gap-junction coupling, the deviation score increases as the coupling increases, suggesting gap-junction-mediated dynamic frustration as in the experiments. Here *T* = 20, *g*_0_ = 1/*T*, and *v*_peak_ = 1. In C and D, 1000 trials are averaged with *τ*_1_ and *τ*_2_ drawn from a Gaussian distribution with mean *T* and standard deviation *T*/10.

Our experimental results suggest that increased coupling between cells may contribute to the destruction of synchronization in MBNNs. To investigate the putative underlying mechanism, we develop a computational model of coupled excitable neurons. Previous theoretical work has identified a particular type of bifurcation, called the saddle-node homoclinic orbit (SNHO) bifurcation, as causing anti-phase desynchonization when two excitable variables are linearly coupled as in gap junction communication^23,24^. The simplest excitable neuron model that exhibits the SNHO bifurcation is the quadratic integrate-and-fire model^25^. In this model, a voltage-like variable *v* undergoes the dynamics *dv*/*dt* = *b* + *v*^2^ until reaching a peak value *v*_peak_, at which point it is reset to a value *v*_reset_. The parameters *b* and *v*_reset_ define a space in which several bifurcations occur (Fig. 4A). The point (*b*,*v*reset) = (0,0), where the saddle-node bifurcation and the saddle homoclinic orbit bifurcation intersect, is the SNHO bifurcation. Points with *v*_reset_ = 0 and sufficiently small *b* > 0 are near the SNHO bifurcation and exhibit spiking dynamics with period^25^ *τ* = *b*^−1/2^ tan^−1^(*b*^−1/2^). We add gap junction coupling with strength *g* between two neurons *v*_1_ and *v*_2_, giving the dynamics

$$\frac{dv_{1}}{dt}=b+v_{1}^{2}+g(v_{2}-v_{1}),\;\frac{dv_{2}}{dt}=b+v_{2}^{2}+g(v_{1}-v_{2}).$$

For weak coupling, *g* ≲ 1/*τ*, the dynamics desynchronize (Fig. 4B, top), whereas for strong coupling *g* ≳ 1/*τ*, the dynamics synchronize (Fig. 4B, bottom), as expected from weak coupling theory^23,26^.

The feature of our experiments distinct from past modeling efforts is the presence of external periodic driving. Therefore, we introduce an external variable *u* that couples to both *v*_1_ and *v*_2_ as

$$\frac{du}{dt}=b_{0}+u^{2},\;\frac{dv_{1}}{dt}=b_{1}+v_{1}^{2}+g(v_{2}-v_{1})+g_{0}(u-v_{1}),\;\frac{dv_{2}}{dt}=b_{2}+v_{2}^{2}+g(v_{1}-v_{2})+g_{0}(u-v_{2}).$$

Here, *u* has period $T=b_{0}^{-1/2}\tan^{-1}(b_{0}^{-1/2})$ and drives *v*_1_ and *v*_2_ with strength *g*_0_. The neurons now have distinct *b*_1_ and *b*_2_ to allow for cell-to-cell heterogeneity. To test whether this model exhibits increased synchronization with increasing driving period for uncoupled neurons (*g* = 0), as in the experiments (Fig. 2B), we draw *b*_1_ and *b*_2_ randomly and calculate the deviation score for different driving periods *T*. Specifically, we draw *b*_*i*_ such that its period $\tau_{i}=b_{i}^{-1/2}\tan^{-1}(b_{i}^{-1/2})$ follows a Gaussian distribution with mean *T* and standard deviation *T*/10. We see in Fig. 4C that as the driving period *T* increases, the deviation score decreases as in Fig. 2B, like $1\sqrt{T}$ as anticipated. To test whether the model exhibits increased deviation score with increasing connectivity for sufficiently large period, as in the experiments (Fig. 2C, *T* = 200 sec), we calculate the deviation score as a function of the cell-cell coupling strength *g*. We see in Fig. 4D that as the coupling strength increases, the deviation score increases, consistent with the experiments. In the model, the reason is that, whereas the external driving alone would tend to synchronize the cells, the cell-cell coupling instead desynchronizes them due to their proximity to the SNHO bifurcation point.

**Figure 5.**
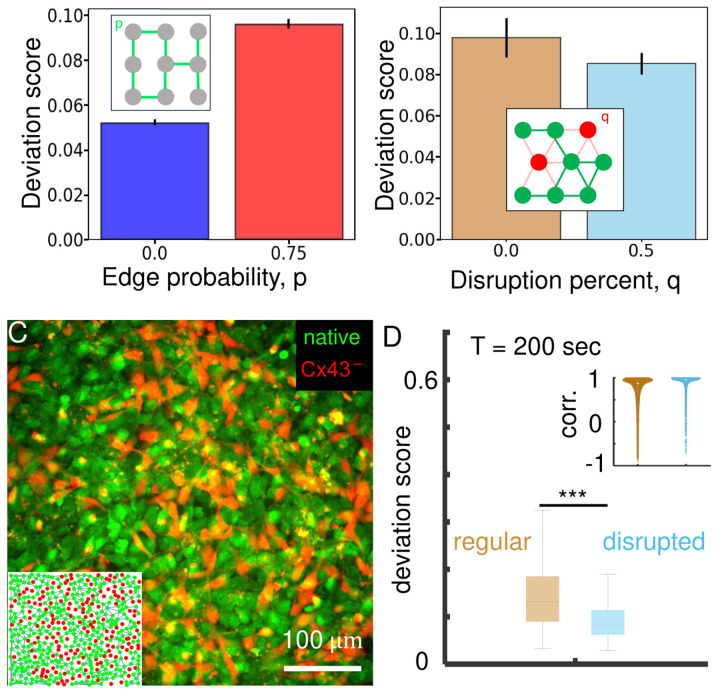
Experimental verification of model predictions demonstrate the nonlinear dynamics of cells may contribute to the dynamic frustration of biological neural networks. (A) Simulation results of the average deviation score for two edge probabilities on a 18×18 square lattice with a period, *T* = 20. A more connected network leads to less synchronized dynamics. 30 trials were run for each *p* and the error bars represent the standard deviation. The inset gives an idea of the network geometry with edges (green) present or not. (B) Simulation results of the average deviation score for two disruption percents on a 10×10 triangular lattice with a period, *T* = 20. A more disrupted network leads to more synchronized dynamics. 15 trials were run for each *q* and the error bars represent the standard deviation. For computational reasons the lattice and trial size are smaller than compared to the square lattice. The inset gives an idea of how disrupted cells affect the network connectivity. (C) Fluorescent image of a KTaR cell monolayer with disrupted intercellular communication. The disrupted multicellular networks consist of 50% native KTaR cells (green, labeled as native in C) and 50% KTaR cells with knocked down expression of connexin 43 (red, labeled as Cx43^−^ in C). Inset: on average each cell has six nearest neighbors where 3 neighbors are communication-deficient Cx43^−^ cells. Neighboring native cells (green dots) are linked by lines. (D) When exposed to slowly varying ATP stimuli (*T* = 200 seconds) cell monolayers with disrupted communication show smaller deviation score compared with regular monolayers formed by native KTaR cells. Inset: the cross-correlation coefficient of neighboring native cells in regular and disrupted KTaR monolayers. In (D) each group includes ~ 700 data points from three replicating experiments and ANOVA test is used to compare the regular and disrupted monolayers. Abbreviations: corr. : cross-correlation coefficient.

To make a more realistic comparison to the MBNNs in our experiments, we have extended our model to a network of neurons on a square lattice. To be comparable in size to experiments, simulations were run on an 18 by 18 square lattice. As above, all neurons were coupled to an external variable acting as a driving force with period *T*. Each simulation lasted for 8 of these periods. Heterogeneity in the lattice was introduced by randomly drawing *b*_*i*_’s for each neuron from a Gaussian distribution with mean *T* and standard deviation *T*/10. Each neuron was connected to a nearest neighbor with edge probability, *p*. If two neurons were connected, their coupling strength was in the weak regime. We see in Fig. 5A that the deviation score increases with edge probability at long period, as in the experiments (Fig. 2C, *T* = 200 sec).

We then use the model to ask whether the dynamic frustration of coupled excitable neurons leads to desynchronization in an confluent monolayer without the controlled structure of the micropatterned grid. To address this question, we consider a coculture of communicating and non-communicating cells, which we have the ability to produce experimentally, described shortly. As we have shown previously^18^, in a confluent monolayer most cells have six nearest neighbors. Therefore, we replace the square lattice in our simulations with the six-neighbor triangular lattice. Simulations were run similarly to the square lattice but instead of an edge probability, each neuron had a probability *q* of being disrupted. If a neuron was disrupted, it would not be connected to any of its six neighbors. We see in Fig. 5B that, due to the desynchronizing effects of the communication, the deviation score is larger in the regular network (consisting entirely of communicating cells) than in the disrupted network (consisting of half communicating, half non-commicating cells).

To test the model prediction in Fig.5B, we constructed a subcloned KTaR cell line possessing disrupted intercellular communication. In particular, we employed a CRISPR-CAS9 strategy (Santa Cruz Biotechnology) to generate a KTaR cell line CX43^−^ with the connexin 43 gene (*gja1*) being knocked out (See SI Fig.5 for characterization of CX43^−^). When mixing native and CX43^−^ cells to form a confluent monolayer, the effective connectivity of the multicellular network is reduced. As an example, in this study we focus on communication-disrupted monolayers consisting of equal amounts of native KTaR cells (green in Fig. 5C) and CX43^−^ cells (red in Fig. 5C). Since most cells have six neighbors, the mixture can be considered a network on a triangular lattice.

We compare the dynamics of regular cell monolayers, which consist of native KTaR cells only, and the dynamics of communication-disrupted monolayers, which consist of a 1:1 ratio of native KTaR cells and CX43^−^ cells. When external temporal signal varies slowly, gap junctional coupling between cells is strong and our model predicts that disrupting communication between cells improves the synchronization. This is indeed the case in our experimental observation. As shown in Fig. 5D, when exposed to ATP stimuli of period *T* =200 seconds, communication-disrupted monolayers have an average deviation score of 0.08, which is nearly half of the deviation score of regular monolayers. Consistently, the nearest-neighbors cells in a communication-disrupted monolayer demonstrate higher cross-correlation coefficients than neighboring cells in a regular monolayer. Together, these experimental results show that the nonlinear excitable dynamics exhibited by neuronal cells plays an important role in contributing to the dynamic frustration of biological neural networks.

## Conclusion and Discussion

While a single cell’s response to temporal signals is regulated by the kinetics of intracellular pathways^27,28,29^, a multicellular system relies on intercellular communications to generate collective sensory responses^30^. In this study, we examine the calcium dynamics of KTaR cells, a neuronal cell line we derived from KNDy neurons within the arcuate nucleus of an adult female mouse. KTaR cells express connexin-43 proteins *in vitro*, which form gap junctions to mediate communications between adjacent cells. We show that the nonlinear, excitable dynamics of KTaR cells are coupled via gap junctions such that the neural network exhibits rich collective dynamics when exposed to rhythmic ATP stimuli. In particular, we combine microfluidics and substrate engineering in order to create micropatterned biological neural networks (MBNNs) whose connectivity can be precisely controlled (Fig.1). To quantify the synchronicity of calcium dynamics in a MBNN excited by temporal signals, we compute (individual-to-population) deviation score and (individual-to-individual) cross-correlation coefficient, which yield consistent results in all our tests. The temporal signals consist of alternate ATP solution, a common neurotransmitter, and pure growth medium, at varying periods.

When exposed to slower temporal signals, as in the case of increasing the period of ATP stimuli, we find that individual nodes in a MBNN become increasingly synchronized (Fig. 2). Because the phenomenon is also present in isolated nodes from MBNNs of zero connectivity (*p* = 0), a putative explanation is that longer period suppress the effects of intercellular heterogeneity^31^. Interestingly, at large period (*T* = 200 seconds), poorly connected neural networks (*p* = 0) outperform highly connected ones (*p* = 0.75) in synchronicity metrics (Fig. 2), suggesting that gap junctions potentially impede communicating cells from synchronization. Indeed, as the period of ATP stimuli increases, we observe an elevation in the expression level of gap junctions, which is accompanied by a concurrent rise in the speed of molecule exchange between cells (Fig. 3). This result is also consistent with previous reports that the information exchange between neuronal cells increases when external stimuli vary at slower rates^18^. Together, our results show that slow temporal signals enhance gap junctional communication between KTaR cells. However the enhanced communication restrains, rather than facilitates synchronized response of a MBNN to external stimuli.

**Figure 6.**
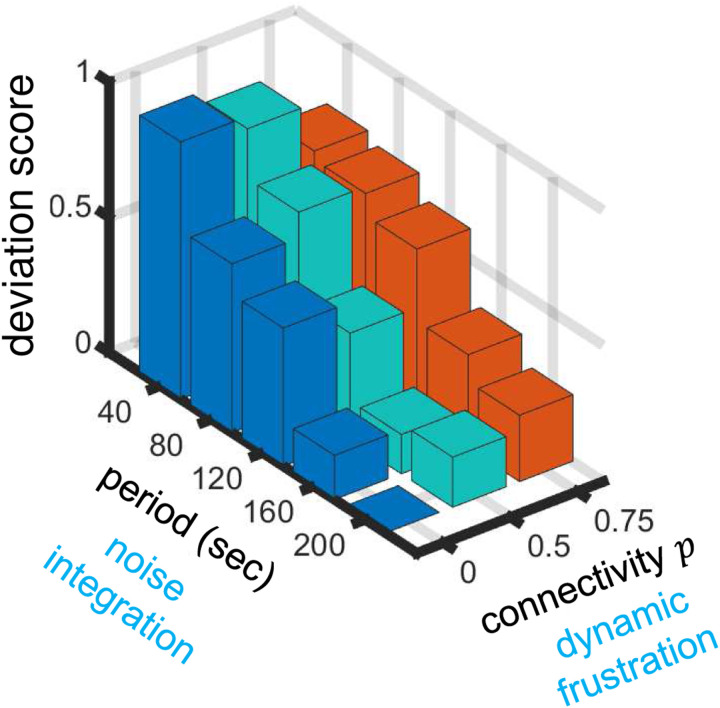
Dynamics of communicating biological neural networks are controlled by external temporal signals (period) and internal connectivity. Our computational model suggests that cell-to-cell deviations decrease with period due to noise integration, and increase with connectivity due to dynamic frustration from the tendency for anti-phase alignment.

In order to understand the role of communication in synchronizing sensory responses of a neural network, we devise a computational model and show that the nonlinear dynamics of individual units depends nontrivially on the interplay between external driving and linear coupling in collective dynamics. The model reproduces the experimental observations that increasing the driving period increases synchronization, and that increasing the coupling between cells decreases synchronization (Fig. 4). The model offers an explanation: coupling desynchronizes cells because the dynamics are near a saddle-node homoclinic orbit (SNHO) bifurcation, which is known to promote anti-phase spiking^24^ and has been implicated in fly wing neurons^23^. The model also makes a prediction—that co-cultures of communicating and non-communicating cells will be more synchronized than a culture of communicating cells—which is confirmed by our experiments (Fig. 5). Taken together, the model suggests that the experimental data can be summarized along two key axes, as shown in Fig. 6: (1) cells synchronize (deviate less) with increasing driving period because longer periods allow for more noise integration; whereas at long periods (2) cells desynchronize (deviate more) with increasing connectivity because coupling promotes anti-phase alignment between neighbors, resulting in dynamic frustration throughout the network.

In mice and other mammals, KNDy neurons are critical for pubertal progression and fertility, and represent hypothalamic neurons capable of pulsatile kisspeptin release. Mechanisms underlying pulse generation are incompletely characterized, and may involve both neuropeptide release and gap junctional communication. Results elucidating cellular synchronization and communication strategies among these neurons will provide insight into these mechanisms.

Biological neural networks are the building block of cognition, learning and intelligence^32,33^. Just like neural networks in computer science, functions of biological neural networks rely on their ability to rewire the inter-neuron interactions^34,35^. Our results show that the property of gap junction mediated neuronal cells interactions can be significantly tuned by an external temporal signal, underscoring the importance of nonlinear neuronal dynamics in controlling collective behaviors. We envision future research to extend the collective sensory responses of micropatterned neural networks to build functional, and adaptive neural circuits.

## Methods

See the SI Appendix for details of cell culture, microscopy and image analysis. The statistical analysis and computer simulations are performed with Matlab (MathWorks^®^).

## Data Availability

The data and code used in this study can be accessed at the following public repositories:
Experimental recordings of calcium dynamics are available at 10.6084/m9.figshare.23298476 The code used to create Figures 4 and 5A–B is available at https://github.com/rwl23/collective_dynamics_of_frustrated_biological_neuron_networks
